# Fire Resistance Evaluation of Concrete Beams and Slabs Incorporating Natural Fiber-Reinforced Polymers

**DOI:** 10.3390/polym15030755

**Published:** 2023-02-02

**Authors:** Venkatesh Kodur, Svetha Venkatachari, Pratik Bhatt, Vasant A. Matsagar, Shamsher Bahadur Singh

**Affiliations:** 1 Department of Civil and Environmental Engineering, Michigan State University, East Lansing, MI 48824, USA; 2 Department of Civil Engineering, National Institute of Technology Puducherry, Karaikal 609609, India; 3 Pacific Structural Forensics, Jersey City, NJ 07310, USA; 4 Department of Civil Engineering, Indian Institute of Technology (IIT) Delhi, Hauz Khas, New Delhi 110016, India; 5 Department of Civil Engineering, Birla Institute of Technology & Science (BITS) Pilani, Vidya Vihar, Pilani 333031, India

**Keywords:** bio-based FRP, fire resistance, externally bonded FRP, near-surface mounted FRP, numerical model, reinforced concrete (RC)

## Abstract

This paper presents a numerical study to evaluate the fire resistance of concrete beams and slabs incorporating natural fiber-reinforced polymers (FRP). A validated finite element model was applied to carry out a series of numerical studies on fire-exposed reinforced concrete (RC) beams and slabs strengthened with conventional and bio-based FRP composites. The model calculates the temperature-dependent moment–curvature relationship for various segments of the member at each time step, which are then used to calculate the moment capacity and deflection of the member. The variables in the beams and slabs include different strengthening techniques (externally bonded FRP and near-surface mounted FRP), different fiber composites, and fire insulation schemes (uninsulated and insulated). The results from the study indicate that the bio-based FRP-strengthened RC members undergo a faster degradation in moment capacity and also experience higher deflections under fire exposure. This leads to a lower fire resistance in RC members with bio-based FRP composites compared to beams and slabs with conventional FRP-strengthened concrete members. The addition of fire insulation to the bio-based FRP-strengthened members can enhance their fire performance and help achieve the required fire resistance ratings for use in building applications. In this study, the NSM CFRP-strengthened RC beams were found to have a fire resistance of 3 h without any fire insulation; however, the bio-based FRP-strengthened beams required a layer of vermiculite–gypsum-based fire insulation material (of about 25 mm) to achieve similar fire resistance.

## 1. Introduction

In recent years, there has been a growing interest to use natural (or bio-based) fiber-reinforced polymer composites in structural applications due to the increasing awareness of the use of renewable, environmentally friendly, and sustainable materials. Different types of natural fibers can be used in the fabrication of FRP composites, namely, animal-based fibers, mineral fibers, and plant-based fibers. Animal-based fibers include silk, wool, hair, and feather. Mineral fibers are obtained from mineral ores and commonly used fibers include asbestos and basalt. Plant-based fibers are obtained from agricultural crops or stubble (waste) and include sisal, flax, ramie, cotton, banana, and hemp.

Natural fiber-based composites that are derived from locally available raw materials such as agricultural waste or stubble form a sustainable alternative to conventional synthetic fiber-reinforced polymer (FRP) composites and their usage in the construction sector can result in the reduction of carbon dioxide (CO_2_) emissions and increased energy savings. Furthermore, plant-based fibers offer numerous advantages over conventional synthetic fibers (such as carbon and glass) such as low-cost, eco-friendly, relatively lightweight, and appreciable strength and stiffness properties [1,2] that make them attractive in various infrastructure applications.

Natural fiber-based FRP composites are currently used in the automobile industry for manufacturing automotive body parts, interior panels, and other non-structural components [3]. In addition, natural fiber-reinforced polymer composites are also used in furniture and packaging industries in the form of decorative panels, packaging cases, etc. [4]. In recent years, the construction industry has been exploring the use of bio-based FRPs in various structural and non-structural components that require low weight and moderate strength. For instance, natural fiber-based polymer composites are used in the manufacture of roof and wall panels, bridge decks for pedestrian footpaths, and other multi-use sheets [5].

There is a large potential for the use of bio-based FRP composites in buildings, especially in repairing and retrofitting structural members with short spans and low to moderate levels of strengthening requirements. There have been some research efforts to develop the needed techniques and guidance for the material and structural design of such members [5,6,7,8,9]. Efforts have been taken to improve the mechanical performance of natural fiber composites to expand their applications. Factors such as fiber and matrix selection, composition, porosity, and manufacturing process that influence the performance of bio-based composites have been evaluated in these studies. However, the above studies only focus on ambient temperature conditions, and the performance of bio-based FRPs at elevated temperatures has not been studied.

For use in buildings, the bio-based FRP composites must satisfy minimum fire resistance requirements prescribed in codes and standards in addition to other fire safety criteria (such as criteria for flame spread, smoke generation, etc.). Currently, there is very limited information on the fire performance of concrete members that incorporate bio-based FRP composites [10,11,12]. For this reason, Michigan State University in collaboration with the Birla Institute of Technology and Science (BITS) Pilani and the Indian Institute of Technology Delhi (IITD) initiated a project to evaluate the fire performance of natural fiber-based composites and generate fire resistance information on structural members incorporating bio-based FRP composites. As part of this project, a detailed literature review of properties needed for characterizing the behavior of bio-based FRP composites at elevated temperatures, as well as testing methods and protocols for developing high-temperature properties was undertaken, which can be found elsewhere [13]. Further, numerical studies were carried out to evaluate the fire performance of bio-based FRP-strengthened members.

This paper presents the fire resistance evaluation of bio-based FRP-strengthened concrete beams and slabs subjected a combination of structural and fire loading. A validated numerical model, originally developed for evaluating the fire resistance of conventional FRP-strengthened members, was extended to evaluate the fire performance of bio-based FRP-strengthened members. Numerical studies were carried out on different bio-based FRP-strengthened concrete members with varying strengthening techniques and insulation schemes. The fire performance of the bio-based FRP composite members was compared with conventional FRP-strengthened concrete members. Practical implications and future research needs for enhancing the fire performance of bio-based FRPs for use in building applications are discussed.

## 2. Need for Fire Resistance in Bio-Based FRP Composites

For strengthening and retrofitting of concrete structures, fiber-reinforced polymer (FRP) composites offer several advantages over conventional construction materials such as concrete and steel [14]. However, unlike concrete and steel, the FRP composites are combustible and have high flammability and poor fire resistance properties that limit their application in buildings. Due to their combustible nature, FRP composites begin to decompose even at low to moderate temperatures in a fire scenario. Moreover, FRP composites undergo significant loss in strength and stiffness when exposed to moderate temperatures of 200 to 300 °C or even lower depending on the composite properties and T_g_ of the resin. For instance, a recent study by Mazzuca et al. [15] found that the shear properties and compressive strength were more affected by temperature than the tensile properties of glass fiber-reinforced polymer laminates [16]. This study reported an 88% reduction in shear modulus, 96% and 67% reduction in compressive strength and modulus, respectively, and 40% and 48% reduction in tensile strength and modulus, respectively, at 200 °C compared to the respective properties of the FRP laminates at ambient conditions.

Further, the interfacial bond strength of FRP composites also degraded drastically at elevated temperatures and this can affect the force transfer between the FRP and concrete. The study by Yu and Kodur [17] showed that the bond strength and modulus of near-surface mounted (NSM) FRP reinforcement degrades significantly in the 20–200 °C temperature range and the FRP possesses negligible bond strength beyond 400 °C. In this study, pull-out tests were carried out to evaluate the effect of temperature on the bond strength of NSM FRP reinforcement. The high-temperature bond tests were performed using a specially designed test setup comprising a tension testing machine and an electric furnace. Through this setup, the bond tests were carried out by heating the NSM FRP system to the desired temperature and then subjecting it to tensile loading. The full details of the test program and results are reported in [17].

Much like conventional FRP composites, bio-based FRP composites also undergo flaming, charring, material degradation, and rapid loss of strength and stiffness properties when exposed to elevated temperatures [10,11,12]. Since bio-based FRP composites incorporate natural fibers (such as rice, corn, hemp fibers, etc.) in place of synthetic fibers (such as carbon or glass), the deterioration in strength and stiffness properties of the composite can be more drastic even at lower temperatures. For the application of bio-based FRP composites in buildings, either for new construction or as repair/strengthening materials, the FRP-incorporated members must satisfy minimum fire resistance requirements prescribed in codes and standards such as IBC 2021 [18] and ACI 216.1-14 [19]. In the case of conventionally reinforced or pre-stressed concrete members, the necessary fire resistance is achieved by providing the minimum member dimensions and a minimum concrete cover to the reinforcement. However, in the case of FRP-incorporated concrete members, the minimum member sizes and cover thickness to satisfy the fire resistance requirements can be different since FRP composites undergo more rapid degradation of both mechanical and bond properties at elevated temperatures as compared to concrete and steel.

Several experimental and numerical studies have been carried out in the past to evaluate the fire resistance of strengthened concrete beams, slabs, and columns incorporating conventional FRP composites [17,20,21,22,23,24,25,26,27]. The experimental studies reported in the literature evaluated the response of FRP-strengthened flexural members with varying FRP reinforcement, insulation schemes, strengthening levels, and fire scenarios through fire resistance tests. The results from these studies have shown that the FRP-strengthened member is vulnerable to faster degradation of strength under fire exposure and thus has lower fire resistance. In many cases, external fire insulation was applied to these members to obtain the required fire resistance ratings. Further, the studies have also indicated that the fire resistance of FRP-strengthened members significantly depends on the type of FRP (glass, carbon, aramid, etc.), FRP properties, strengthening levels, fire severity, and any insulation provided on the member.

In the numerical studies, macroscopic or microscopic finite element-based models were used to predict the performance of FRP-strengthened concrete members under fire conditions. Hawileh et al. [28] developed a finite element (FE)-based model in ANSYS to evaluate the thermal and structural response of FRP-strengthened reinforced concrete (RC) T-beams exposed to fire. The model accounted for the temperature-dependent properties of concrete, steel reinforcement, FRP, and insulation material. However, the bond–slip at the FRP-concrete interface was not modeled in the study. Kodur and Ahmed [27] developed a macroscopic FE model in FORTRAN to trace the response of FRP-strengthened RC beams under combined structural and fire loading. The model uses secant stiffness, evaluated using the temperature-dependent sectional moment–curvature relationship, to compute the moment capacity and deflection in the beams. The model was further modified by Kodur and Bhatt [21] to incorporate the softening behavior of concrete and temperature-induced bond degradation between concrete and FRP reinforcement.

Dai et al. [24] developed a 3-D FE model in ABAQUS to evaluate the fire response of FRP-strengthened RC beams. The model explicitly accounted for the nonlinear bond–slip behavior at the FRP–concrete interface as well as the steel rebar–concrete interface using a cohesive zone model and spring elements. Dong et al. [29] developed an FE-based model in ANSYS to predict the performance of FRP-strengthened structural members. The model accounted for the bond degradation between FRP and concrete by considering a temperature-dependent reduction factor while evaluating the strength degradation in the FRP reinforcement. In the case of bio-based FRP composites, very limited information is available in the literature on the fire performance of these members. Even the material property data for bio-based FRPs are limited to very few studies at ambient temperature conditions and not much information is available regarding the variation of properties at elevated temperatures.

For evaluating the fire resistance of FRP-strengthened members, not many empirical relationships or design charts are available [14,30], and even these are limited to conventional FRP-strengthened members and cannot be extended directly to bio-based FRP-strengthened members. Standard fire tests can be used to evaluate the fire resistance of bio-based FRP composite members; however, only very few parameters can be evaluated in these tests and such tests are expensive to undertake. Alternatively, numerical methods can be applied for evaluating the fire resistance of bio-based FRP-strengthened members. In these methods, various governing factors such as strengthening schemes, fire scenarios, load and restraint conditions, FRP and insulation properties, debonding of FRP, as well as realistic failure limit states can be accounted for. The numerical model used in this study for evaluating the fire resistance of bio-based FRP-strengthened members is presented in the next section.

## 3. Numerical Model

To assess the fire resistance of reinforced concrete (RC) members with natural fiber-reinforced polymers, a previously developed macroscopic finite element-based model was utilized. The selected numerical model was originally developed by Kodur and Ahmed [27] using the FORTRAN program and further extended by Kodur and Bhatt [21] to include the effects of the softening behavior of concrete and temperature-induced bond degradation between FRP and concrete. The numerical model has been used previously to evaluate the fire resistance of conventional FRP-strengthened RC beams and slabs, both with externally bonded (EB) FRP reinforcement and near-surface mounted (NSM) reinforcement. The model predictions have shown good agreement with that of test data and complete details of the validation studies can be found in [17,21,27]. In the present study, the model will be applied to examine the fire performance of bio-based FRP-strengthened RC members by using the appropriate material properties, discretization, and failure limits for these natural fiber-based composite members.

### 3.1. Analysis Procedure

**Figure 1:**
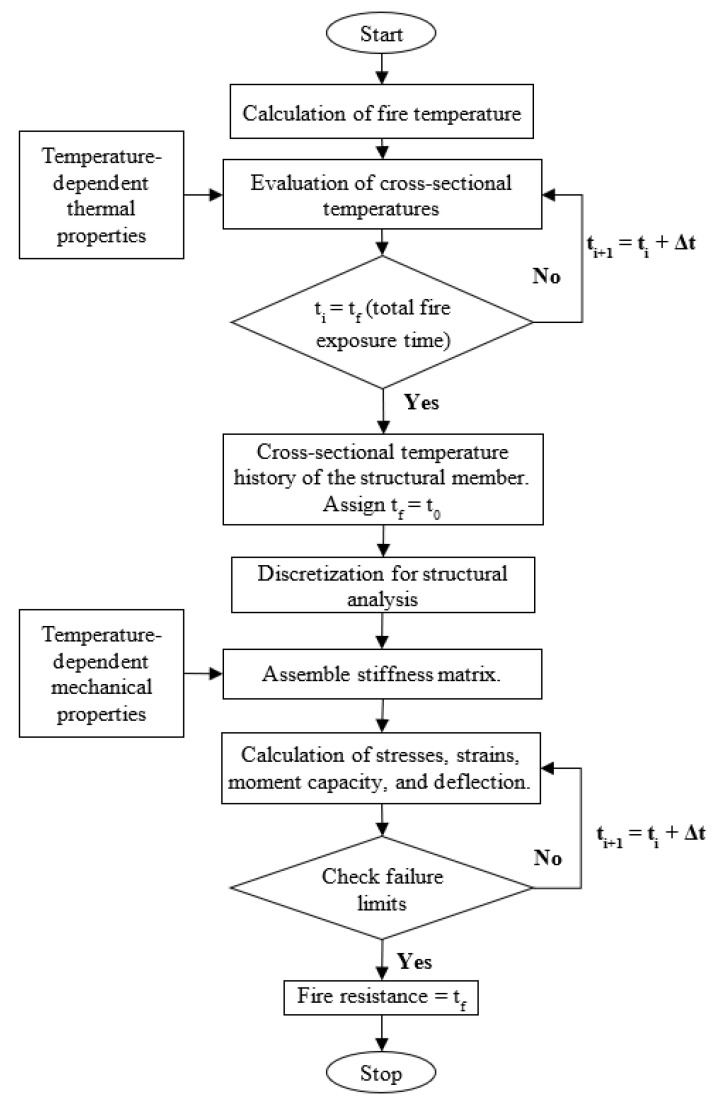
Flowchart outlining the steps used in the numerical model for evaluating the fire resistance of FRP-strengthened RC members.

The numerical model for tracing the fire response of the FRP-strengthened RC beams and slabs follows a sequential procedure, starting with a thermal analysis followed by a structural analysis. Figure 1 shows the steps outlining the procedure used in the analysis.

For the fire resistance calculations, the FRP-strengthened member was discretized into several segments along the span of the member, and the cross-section at the center of each segment is divided into several rectangular elements. The temperature rise in each element, at a given cross-section, was calculated by carrying out a 2-D heat transfer analysis. The transfer of heat from the fire zone to the boundaries of the concrete member (or insulation) was modeled using convection and radiation boundary conditions. The transfer of heat within the cross-section of the concrete member is mainly through conduction. The emissivity coefficient was taken as 0.8 as per Eurocode 1–2 provisions [31] while specifying the radiative boundary conditions on the fire-exposed surface of the member. The convection coefficient on the fire-exposed surface was taken as 25 W/m^2^K as per Eurocode 1–2 provisions [31] for the standard temperature–time curve. The thermal analysis takes into account the temperature-dependent thermal properties of the concrete, steel rebar, FRP, and applied fire insulation. The cross-sectional temperatures (in the FRP, steel rebar, and concrete elements) obtained from the thermal analysis are provided as input to the structural model to evaluate the reduction in strength and stiffness properties of the constituent materials.

The structural analysis was carried out in incremental time steps. At each time increment, moment–curvature relations are generated for the various segments of the member by applying the conditions for force equilibrium and strain compatibility. These time-dependent moment–curvature relationships were used to carry out a stiffness analysis to obtain the moment and deflection at different segments in the FRP-strengthened member. The temperature-dependent mechanical properties of concrete, steel rebar, and FRP were supplied as input to the structural model. The bond–slip between the FRP reinforcement and concrete was not accounted for in the numerical analysis. Owing to the lower strength and stiffness of bio-based FRP composites compared to conventional FRP composites, as well as more rapid degradation of these properties with temperature, the bio-based FRP-strengthened member fails at earlier times due to loss of capacity before significant slip occurs. Moreover, accounting for the bond–slip of FRP reinforcement requires detailed information on the variation of bond properties of bio-based FRP composites with temperature, which is not available in the current literature. For these reasons, the present study neglects any bond–slip between the FRP reinforcement and concrete.

At each time step, different output parameters including cross-sectional temperatures, stresses and strains, moment capacity of the strengthened member, and mid-span deflection are obtained from the analysis. These response parameters were compared with the failure limits prescribed in ASTM E119 [32] to determine the failure time (or fire resistance) of the member. The failure limit states considered for this study include the critical temperature of conventional steel reinforcement, as well as more realistic strength- and displacement-based limit states. Further details of the numerical model along with the validation studies can be found in [17,21,27].

### 3.2. Material Properties

For evaluating the fire resistance of natural FRP-strengthened members, the thermal and mechanical properties of the constituent materials, i.e., concrete, steel reinforcement, FRP, and fire insulation along with the variation of these properties with temperature are required. For the thermal analysis, the properties of interest include density, specific heat, and thermal conductivity. For concrete and steel rebar, the temperature-dependent thermal property variations were obtained from Eurocode 2 [33]. However, very limited data is available on the thermal properties of FRP composites at room and elevated temperatures, and even this information is restricted to conventional FRP composites. Due to the lack of data in the literature, the thermal property relationships established by Griff et al. [34] for conventional FRPs were used for bio-based FRPs as well. It should be noted that there is no complete agreement in the literature on the variation of thermal properties such as thermal conductivity and specific heat of FRP, and hence there may be some variation in the results due to not considering the appropriate thermal properties of the FRP. However, since the size of the FRP reinforcement is much smaller compared to that of the concrete cross-section, the thermal properties of the FRP tend to have minor influences on the evaluation of fire resistance of the strengthened concrete member [30]. For the fire insulation, sprayed fire-resistive material (SFRM) was used and the thermal properties of the insulation were obtained from the material property tests reported by Kodur and Shakya [35].

For carrying out the structural analysis, high-temperature stress–strain relationships of concrete and steel rebar specified in Eurocode 2 [33] were used in the model. For conventional FRP, the degradation in strength and stiffness properties specified by Bisby et al. [36] were utilized. Limited information is available regarding the strength and stiffness properties of natural fiber-based FRPs at room temperature and there is no reliable data on the variation of these properties at elevated temperatures. In general, bio-based FRPs have lower strength and stiffness in comparison to conventional FRPs such as glass fiber-reinforced polymer (GFRP) or carbon fiber-reinforced polymer (CFRP) [10,11]. Since bio-based FRPs incorporate plant fibers, the drop in strength and stiffness will occur early (lower temperature rise) and at a more rapid pace than with conventional FRPs. Hence, for this study, a linear degradation in strength and stiffness was assumed for the bio-based FRPs, where the properties begin to degrade at 100 °C and at 200 °C the bio-based FRP loses most (more than 90%) of its strength/stiffness. Studies in the literature indicate that the bio-based FRP composites tend to decompose beyond 230 °C [37] and hence, the assumed behavior will provide conservative fire resistance predictions.

**Figure 2:**
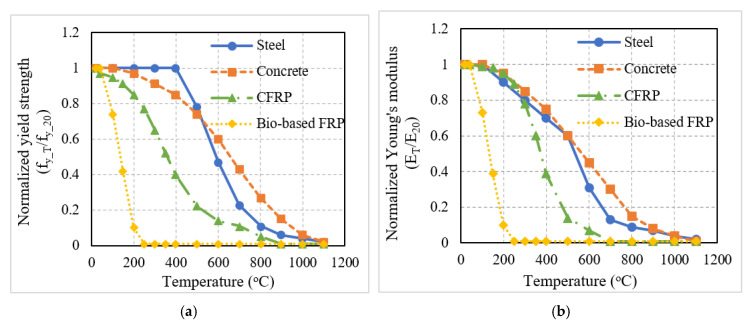
Variation of normalized yield strength and elastic modulus of steel, concrete, conventional FRP (CFRP), and bio-based FRP. (**a**) Yield strength; (**b**) elastic modulus.

Figure 2 shows the degradation of strength and elastic modulus of concrete, steel rebar, conventional FRP (CFRP), and bio-based FRP. It can be seen that the reduction in strength and stiffness of the FRP material was more rapid when compared to concrete and reinforcing steel. Bio-based FRP experienced an even more drastic reduction as compared to conventional FRP. The critical temperature for the conventional FRP composite is defined as the temperature at which the FRP loses 50% of its strength [16]. For instance, the critical temperature for CFRP and GFRP, used in strengthening applications are 250 °C and 325 °C, respectively [16]. On the other hand, for the bio-based FRP assumed in this study, the critical temperature was 150 °C.

Additionally, a linear elastic stress–strain behavior was assumed for the FRP material at room and elevated temperatures, and this is as per the trends shown in previous tests. Further, the thermal expansion coefficient for concrete and reinforcing steel was taken from Eurocode 2 [33]. For bio-based FRP, no data are available in the literature regarding thermal expansion and hence, the assumptions made for conventional FRP were applied for bio-based FRP as well. In the case of conventional FRP, the coefficient of thermal expansion in the longitudinal direction is very small ($-0.09\times 10^{-9}/$°C), and the thermal strain in the longitudinal direction is considered negligible [21]. Since the natural fibers are assumed to be oriented along the longitudinal direction of the concrete member, the thermal strain in the bio-based FRP reinforcement was also considered to be negligible for this study. The material properties of concrete, steel rebar, insulation, and FRP reinforcement used in the numerical model are summarized in Table 1.

### 3.3. Modeling Assumptions

In the numerical model, the following assumptions were made for evaluating the fire resistance of FRP-strengthened RC members:

1. For the generation of moment–curvature relationships, plane sections were assumed to remain plane before and after bending.
2. Only the flexural limit state was considered while evaluating the failure of the beam (or slab) and the shear limit state was ignored.
3. The bond–slip between the steel reinforcement and concrete and that between the FRP reinforcement and concrete were not considered in the model.
4. The FRP reinforcement was considered to exhibit a linear stress–strain response at elevated temperatures up to failure.
5. The thermal properties of the bio-based FRP reinforcement were considered to be the same as those of conventional FRP reinforcement.

## 4. Numerical Studies

### 4.1. General

The numerical model described in the previous section was applied to evaluate the fire resistance of FRP-strengthened RC flexural members (beams and slabs) with different types of fibers (conventional and bio-based), strengthening types (NSM and EB), and fire protection schemes (with and without insulation). The members with conventional FRP strengthening were used as the base case and the fire performance of the natural fiber-reinforced RC members was compared against the conventional ones.

### 4.2. Details of the Flexural Members and Parameters Considered for the Numerical Study

**Figure 3:**
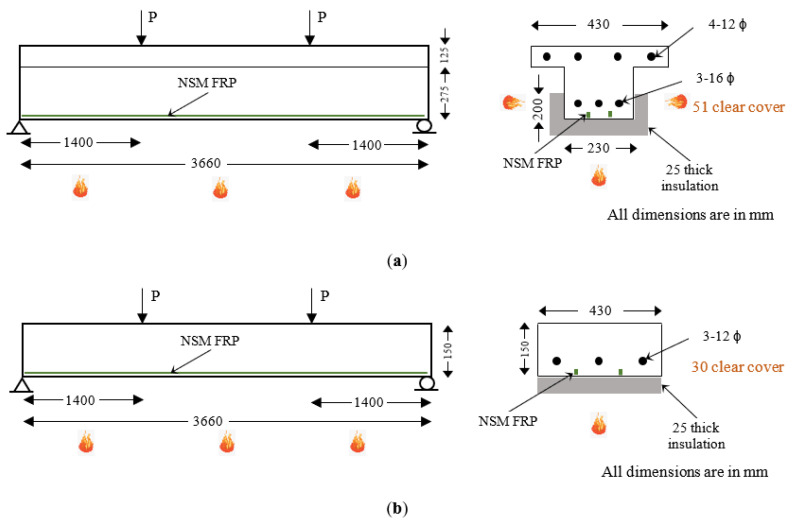
Layout and geometric details of RC members strengthened with NSM-type FRP reinforcement. (**a**) RC T-beam strengthened with NSM FRP reinforcement; (**b**) RC slab strengthened with NSM FRP reinforcement.

**Figure 4:**
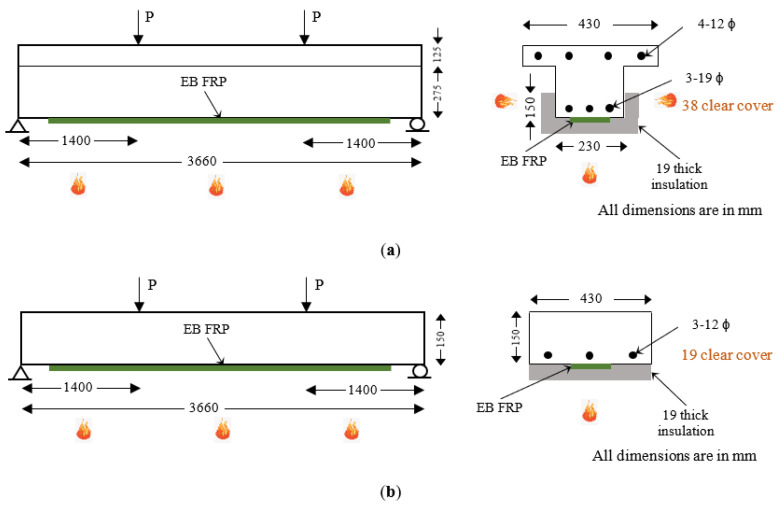
Layout and geometric details of RC members strengthened with EB-type FRP reinforcement. (**a**) RC T-beam strengthened with EB FRP reinforcement; (**b**) RC slab strengthened with EB FRP reinforcement.

The numerical studies are presented for two broad cases: members with NSM FRP reinforcement and members with externally bonded (EB) FRP reinforcement. Figure 3 and Figure 4 show the geometric configuration of the T-beams and slabs considered in this numerical study. In each case, four different configurations of the above-mentioned T-beams and slabs were analyzed to evaluate the relative fire performance of the flexural members. Table 2 and Table 3 present the details of T-beams and slabs strengthened with NSM FRP reinforcement and EB FRP reinforcement, respectively.

Beams B1 and B2 were provided with two NSM CFRP strips with a cross-sectional size of 13.5 mm × 4.5 mm, whereas beams B3 and B4 were provided with five NSM bio-based FRP strips 20 mm × 10 mm in size each. Additionally, beams B1 and B3 were uninsulated, whereas beams B2 and B4 were provided with 25 mm U-shaped SFRM-type fire insulation (Figure 3). Similar to the T-beams, two slabs (S1 and S2) were provided with two CFRP strips each while the other two slabs (S3 and S4) are each provided with four bio-based FRP strips (Table 2). In addition, the insulation scheme was varied such that slabs S1 and S3 were uninsulated whereas slabs S2 and S4 were insulated with 25 mm fireproofing material near the bottom surface.

The NSM FRP reinforcement was selected to achieve an increase in moment capacity of 50% in CFRP-strengthened members, whereas only a 15% increase in moment capacity was achieved in bio-based FRP-strengthened members. This is because of the limited space available between the steel reinforcement and the bottom surface of the concrete member that hinders the placement of additional (or larger) bio-based FRP strips, which in turn limits the potential to increase the moment capacity of the bio-based FRP-strengthened members beyond a certain extent.

For members with EB FRP reinforcement, the FRP type and insulation scheme were varied similar to the NSM FRP beams and slabs as shown in Table 3. CFRP-type strengthening was provided on two beams (B5 and B6) and two slabs (S5 and S6), whereas bio-based FRP-type strengthening was applied on the other two beams (B7 and B8) and two slabs (S7 and S8). The EB FRP reinforcement was selected to achieve a 25% increase in moment capacity in all beams and slabs considered in this case. No fire protection was provided on four of the beams and slabs (B5, B7, S5, and S7). The other four members (B6, B8, S6, and S8) were provided with 19 mm fire insulation as shown in Figure 4.

Each beam and slab were divided into 20 segments along the length of the member. The cross-section of each beam was discretized into 10 mm × 10 mm elements, whereas the cross-section of each slab was discretized into 10 mm × 5 mm elements. For structural members strengthened with EB FRP reinforcement, the CFRP strips were discretized into 10 mm × 1 mm elements, whereas the bio-based FRP strips were discretized into 10 mm × 10 mm elements. The mesh used in the structural analysis was the same as that used in the thermal analysis.

**Figure 5:**
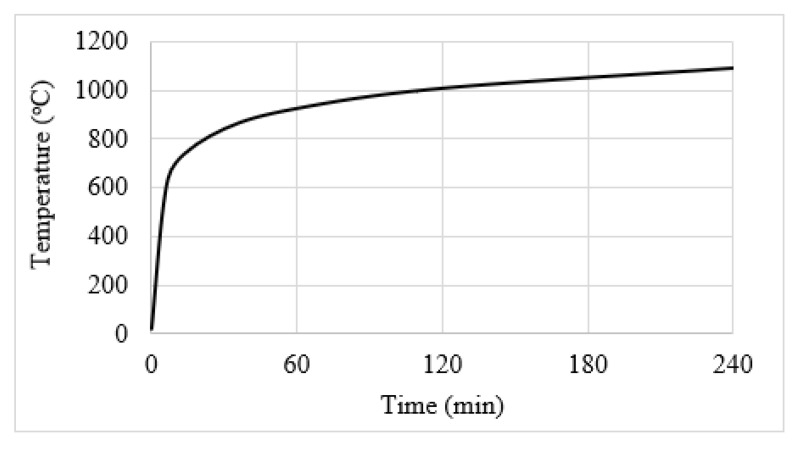
ASTM E119 fire exposure scenario used in the numerical studies.

For the numerical analyses, the tensile strength and elastic modulus were set as 2.51 GPa and 139.6 GPa, respectively, for the NSM CFRP strips (62% by volume of carbon fiber in epoxy matrix) [38] and as 1.17 GPa and 96.5 GPa, respectively, for the EB CFRP strips (92% by weight of carbon fiber in epoxy matrix) [22]. For the bio-based FRP, hemp fiber-based polymer composite strips (40% by weight of hemp fiber in polypropylene matrix) with a tensile strength of 52 MPa and elastic modulus of 6.8 GPa were considered in all the analyses. These values were obtained based on the studies carried out by [39]. The flexural members were simply supported and subjected to two concentrated loads. The ASTM E119 [32] standard fire exposure scenario (shown in Figure 5) was considered for all cases and the analyses are carried out for 240 min of exposure or until failure, whichever occurred first. The beams were exposed to fire along the two side surfaces and the bottom surface, whereas the slabs are exposed to fire only from the bottom surface.

### 4.3. Fire Resistance of Members with NSM FRP Reinforcement

**Figure 6:**
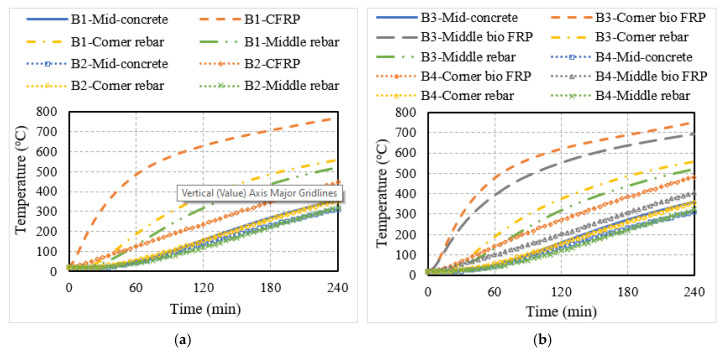

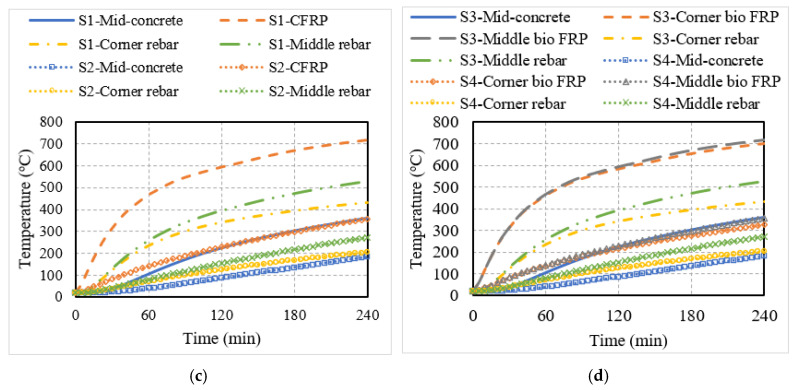
Thermal response of RC members strengthened with conventional or bio-based NSM FRP reinforcement. (**a**) NSM CFRP-strengthened T-beam; (**b**) NSM bio-based FRP-strengthened T-beam; (**c**) NSM CFRP-strengthened slab; (**d**) NSM bio-based FRP-strengthened slab.

The fire response of the T-beams and slabs strengthened with either conventional or bio-based NSM FRP reinforcement (summarized in Table 2) is discussed in this section. The thermal response of the FRP-strengthened members is illustrated in Figure 6 by plotting the temperatures at the mid-depth of the concrete section in the FRP reinforcement, and as well as in the steel reinforcement as a function of fire exposure time. It can be seen that the temperatures at different points in the cross-section of the insulated T-beams and slabs were much lower as compared to the corresponding points in the uninsulated members for the entire duration of fire exposure. In the case of uninsulated members, the NSM FRP strips (both conventional and bio-based FRP), which are located close to the surface of the member, experienced a rapid increase in temperature with increasing fire exposure time.

The time taken to exceed the critical temperature of 150 °C in the bio-based FRP-strengthened members was around 20 min for uninsulated members as compared to 60 min for fire-insulated members. However, even with the presence of similar insulation thickness, the time taken to exceed the critical temperature in bio-based FRP-strengthened members was significantly lower than in synthetic CFRP-strengthened members, which took 120 min to exceed the critical temperature of 250 °C. This indicates that the bio-based FRP-strengthened member is likely to lose the majority of its strengthening beyond 60 min of fire exposure even when provided with 25 mm of fire protection. Overall, the temperatures in the slab were slightly lower than in T-beams. This can be attributed to the three-side heating in the case of the T-beams compared to slabs, which were exposed to fire only from the bottom surface. Further, the temperatures in the steel rebars remained below the critical steel temperature of 593 °C for the entire duration of the fire exposure in all the members, indicating no imminent failure of the beams/slabs.

**Figure 7:**
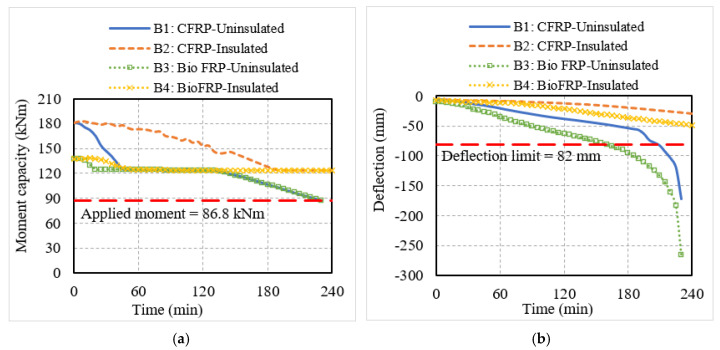
Structural response of T-beams strengthened with conventional or bio-based NSM FRP reinforcement. (**a**) Moment capacity; (**b**) mid-span deflection.

**Figure 8:**
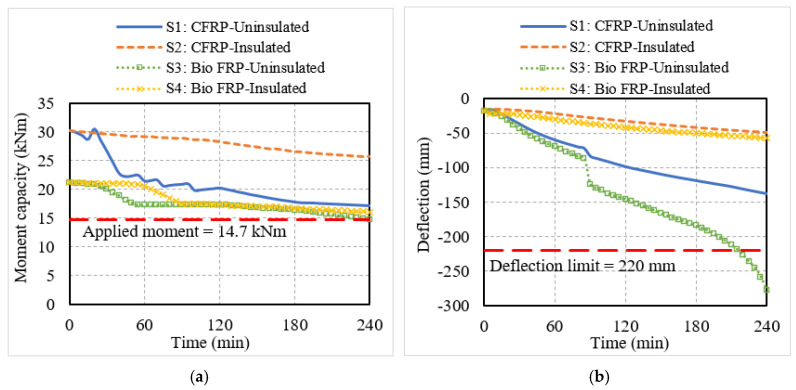
Structural response of slabs strengthened with conventional or bio-based NSM FRP reinforcement. (**a**) Moment capacity; (**b**) mid-span deflection.

The structural response of the NSM FRP-strengthened beams and slabs was monitored through the degradation in moment capacity and mid-span deflection of the structural members, which are shown in Figure 7 and Figure 8. In the flexural members with no fire protection, the moment capacity decreased rapidly in the initial phase of fire exposure. This is because the NSM strips experienced a significant rise in temperature due to their proximity to the fire-exposed surfaces of the flexural members. In comparison to the CFRP-strengthened members, the moment capacity degradation in the bio-based FRP members was more rapid owing to the faster degradation of the strength and stiffness properties of the bio-based FRP with temperature. This can be seen from the response of beam B3 and slab S3, which lost their entire strengthening contribution at 20 min and 50 min, respectively, as compared to the CFRP beam B1 and slab S1 which lost their strengthening contribution at 90 min and 120 min, respectively. After losing their strengthening, the flexural members behaved as conventional RC members without much contribution from the CFRP or bio-based FRP reinforcement. With a further increase in fire temperature, the moment capacity of the uninsulated members began to gradually degrade but did not drop below the corresponding applied moments in the beams and slabs, indicating that the failure did not occur due to strength criteria.

Similar trends were observed in the deflection response of the uninsulated flexural members. It can be seen from Figure 7 and Figure 8 that the deflections in beams B1 and B3 and slabs S1 and S3 were increasing rapidly from the start of the fire exposure time. The bio-based FRP-strengthened members experienced higher deflections compared to the conventional CFRP-strengthened members due to the faster degradation of stiffness of the bio-based FRP with the rise in temperature. The deflections in all the uninsulated members except slab S1 exceeded the deflection limit state, indicating deflection-based failure. Even though the moment capacity and deflection degraded rapidly, slab S1, owing to its high initial strengthening, could withstand the fire exposure for the entire 240 min without undergoing failure.

The fire-insulated conventional and bio-based NSM FRP-strengthened members performed better under fire exposure. The fire insulation prevented the FRP reinforcement from experiencing a rapid increase in temperature, and hence the flexural members can retain their strengthening levels for a longer duration of fire exposure. This can be seen from the more gradual decrease in moment capacity and deflection of the insulated members in comparison to the uninsulated members in Figure 7 and Figure 8. Although the presence of fire insulation delayed the loss of strengthening in the bio-based FRP-strengthened members, beam B4 and slab S4 lost their strengthening faster at 55 min and 90 min, respectively, compared to the CFRP-strengthened members, which did not lose their strengthening until 180 min or longer. This is because the initial strengthening levels were higher in the case of CFRP-strengthened members and in addition, the bio-based FRP tended to lose its strength and stiffness faster compared to CFRP. The moment capacity and deflection of the insulated flexural members were above the respective limits and hence, these members did not undergo failure within the 240 min of fire exposure.

Overall, the results indicate that while the NSM CFRP-strengthened RC beam can provide three hours of fire resistance under standard fire exposure even without the presence of any fire insulation, the bio-based FRP-strengthened beam requires a layer of fire insulation to achieve similar fire resistance. Likewise, the uninsulated bio-based FRP slab undergoes significantly higher deflections and a faster decrease in moment capacity, and fire insulation is needed in bio-based FRP-strengthened slabs to enhance their fire response and provide similar performance as that of CFRP-strengthened slabs.

### 4.4. Fire Resistance of Members with EB FRP Reinforcement

**Figure 9:**
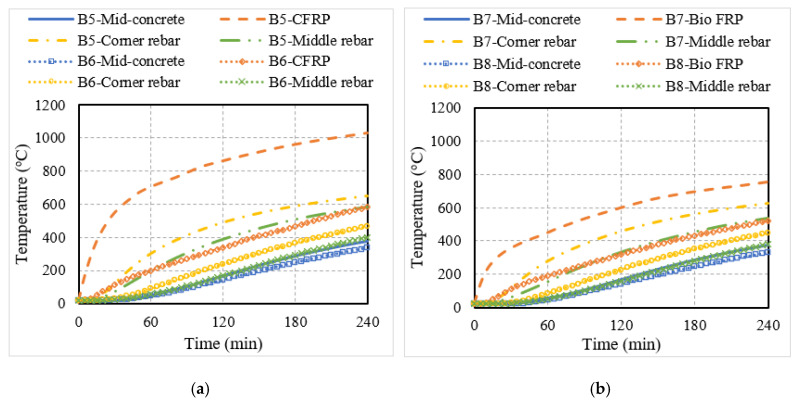

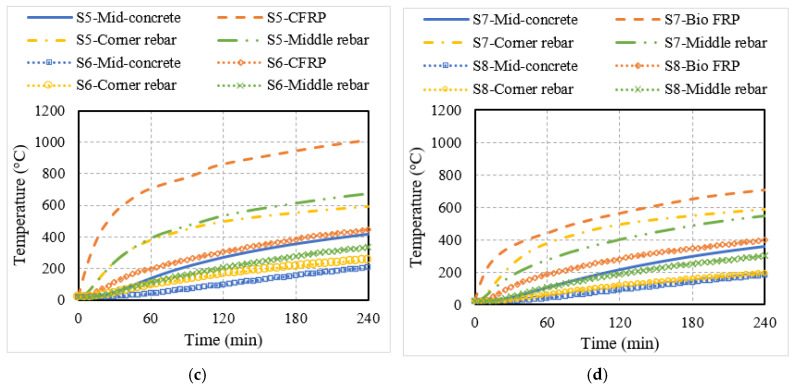
Thermal response of RC members strengthened with conventional or bio-based EB FRP reinforcement. (**a**) EB CFRP-strengthened T-beam; (**b**) EB bio-based FRP-strengthened T-beam; (**c**) EB CFRP-strengthened slab; (**d**) EB bio-based FRP-strengthened slab.

To investigate the fire performance of RC members strengthened with externally bonded (EB) FRP reinforcements, eight different configurations of beams and slabs were analyzed. The temperatures obtained from the thermal analysis for concrete (at mid-depth), steel rebar, and FRP are plotted for the different flexural members in Figure 9. In comparison to the NSM FRP-strengthened members, the EB FRP reinforcement experienced higher temperatures since the FRP is externally applied to the member in the latter case and hence, is in closer proximity to the fire exposure. For this reason, the EB FRP reinforcement reached the critical temperature more quickly than the NSM reinforcement. For instance, in the case of the bio-based FRP-strengthened members, the time taken to reach the critical temperature was 5 min for uninsulated members and 45 min for insulated members.

From Figure 9, it can also be seen that the temperatures in the bio-based FRP reinforcement were lower than that of the CFRP reinforcement, especially in the case of uninsulated members. This is because the temperature in the FRP was measured at the mid-height and the thickness of the bio-based FRP (10 mm) which is larger than the CFRP strip (1 mm), which in turn results in a slower transfer of heat in the bio-based FRP. However, in uninsulated members, the FRP is likely to disintegrate early during fire exposure since they are combustible and will not contribute much to the fire resistance of the member. Further, the steel rebar temperatures increased beyond the critical temperature of 593 °C in the later stages of fire exposure (around 180 min) in the uninsulated flexural members, indicating failure based on the critical temperature limit state. However, for a more realistic evaluation of fire performance, the structural response of the FRP-strengthened RC members was traced under fire exposure.

**Figure 10:**
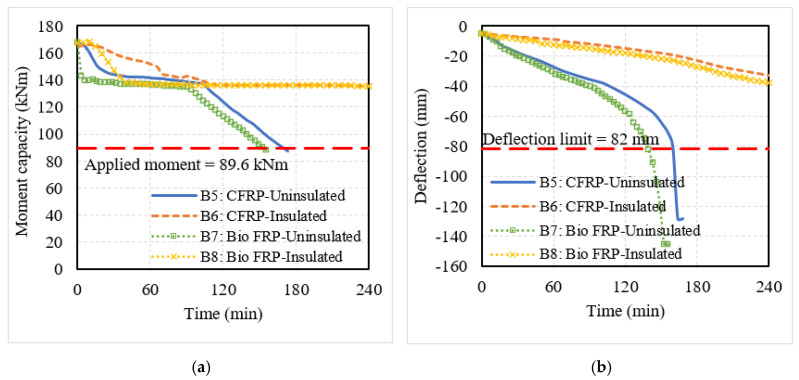
Structural response of T-beams strengthened with EB FRP reinforcement. (**a**) Moment capacity; (**b**) mid-span deflection.

**Figure 11:**
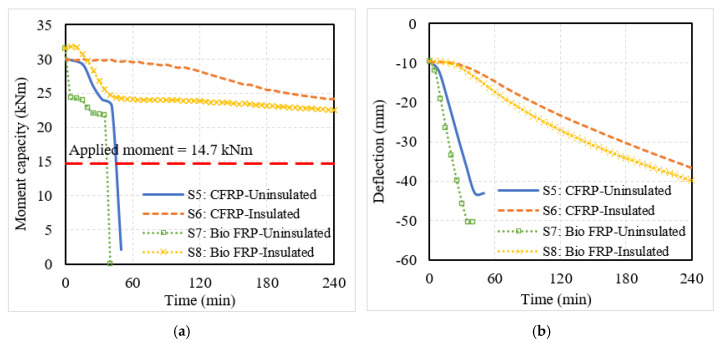
Structural response of slabs strengthened with conventional or bio-based EB FRP reinforcement. (**a**) Moment capacity; (**b**) mid-span deflection.

The structural response was evaluated through the degradation in moment capacity and mid-span deflections of the EB FRP-strengthened RC members, which are plotted in Figure 10 and Figure 11. Similar to the behavior of NSM FRP-strengthened members, the uninsulated EB FRP-strengthened members showed a rapid degradation in moment capacity and increased deflections from the beginning of fire exposure. However, since the FRP reinforcement was externally applied on the surface of the members, the capacity degradation and increase in deflection were more drastic as compared to NSM FRP-strengthened members where the FRP reinforcement was located inside the concrete cross-section. The failure in the FRP-strengthened RC beams B5 and B7 occurred at 165 min and 140 min, respectively, when the deflection in these members exceeded the deflection limit of 82 mm. On the other hand, the FRP-strengthened slabs S5 and S7 failed due to their moment capacity dropping below the applied moment at 45 min and 35 min, respectively.

When fire insulation was applied to the flexural members, the fire performance of both beams and slabs improved significantly. The FRP-strengthened RC members (B6, B8, S6, and S8) experienced a slower degradation in moment capacity and a gradual increase in mid-span deflections as seen in Figure 10 and Figure 11. The moment and deflection values in these flexural members did not exceed the corresponding limiting values and hence, did not undergo failure. Similar to the NSM bio-based FRP-strengthened members, the EB bio-based FRP also showed poorer fire performance as compared to the CFRP-strengthened members. Since the externally bonded bio-based FRP was more easily exposed to fire due to its location, the performance of these members was severely affected under fire conditions and fire insulation is needed in all cases to achieve adequate fire resistance for use in buildings.

It should be noted that the results obtained from the numerical analyses were mainly qualitative and some differences are expected due to the differences between the actual temperature dependencies of the material properties of the FRP reinforcement and those considered in the numerical model. Further, the results obtained are influenced by not considering the appropriate bond stress–slip relations.

## 5. Practical Implications

This study primarily focused on evaluating the fire resistance of bio-based FRP-strengthened concrete members. FRP materials, as discussed before, have numerous advantages that make them attractive for structural applications; however, the main drawbacks of using these materials in buildings are their high flammability, large smoke development, and poor fire resistance properties. Bio-based FRP composites also face the same issues. Further, natural fibers have lower initial strength and stiffness as compared to synthetic fibers, and therefore the fire resistance of bio-based FRP composite members is even lower than the conventional FRP-strengthened members. From the numerical studies, it can be inferred that the fire resistance of the FRP-strengthened members significantly depends on the material properties of the FRP at elevated temperatures. However, there is a lack of reliable material property data for bio-based FRP under room and elevated temperature conditions. This stems from the limited work that has been carried out in this area and the lack of standardized test methods and procedures to characterize the high-temperature properties of these FRP materials. Further research is needed to develop temperature-dependent property data and material models for bio-based FRP composites. The numerical model presented in this study can then be updated with the available material property data to evaluate the required FRP reinforcement, member sizes, cover thicknesses, and fire insulation to achieve the necessary fire resistance ratings for structural applications.

Bio-based FRP were intended to be developed as a retrofitting material for applications with low (or moderate) strengthening requirements such as rural infrastructure. In such applications, locally sourced raw materials (e.g., agricultural waste) can be utilized in developing the FRP material which can lead to cost savings as well as reduced carbon emissions in the construction sector. From the numerical studies carried out in this paper, it is inferred that to achieve reasonable strengthening levels in the flexural members, a larger thickness (or a higher number) of bio-based FRP reinforcement is required in comparison to the conventional CFRP reinforcement. In addition, the insulation thickness required in the case of bio-based FRP-strengthened members for achieving similar fire performance as that of conventional FRP members is higher. This can result in practical challenges such as the need for larger cover thickness, placement of FRP reinforcement, debonding of FRP and insulation, etc. Further research is needed to overcome these challenges in realizing the potential application of bio-based FRP composites in buildings. Further, fire resistance tests of bio-based FRP composite members need to be carried out to generate more test data to validate the predictions from the numerical model.

## 6. Conclusions

This study evaluated the fire performance of bio-based FRP-strengthened RC flexural members under the combined effects of structural and fire loading. A set of numerical analyses was carried out on FRP-strengthened beams and slabs with varying strengthening techniques and insulation schemes. The fire resistance of the bio-based FRP composite members was compared with conventional FRP-strengthened concrete members. The results obtained from the numerical studies led to the following conclusions on the fire performance of natural fiber-reinforced polymer (FRP) composite beams and slabs:

1. The fire performance of bio-based FRP-strengthened concrete members was lower than that of a conventional CFRP-strengthened member with similar design parameters. This is due to the lower initial strength and stiffness properties of the bio-based FRP and the faster degradation of its mechanical properties with a rise in temperature.
2. Uninsulated NSM bio-based FRP-strengthened members experienced higher deflections and a faster degradation in moment capacity as compared to the conventional CFRP counterparts. While the NSM CFRP-strengthened RC beams can achieve up to 3 h of fire resistance without any fire insulation, the bio-based FRP-strengthened beams required a layer of fire insulation (of about 25 mm) to achieve similar fire resistance.
3. Uninsulated EB bio-based FRP-strengthened RC members undergo severe performance degradation under fire conditions due to direct exposure of the FRP reinforcement to fire. Hence, fire insulation is always needed when the bio-based FRP reinforcement is to be externally applied to an RC member to achieve the required fire resistance ratings as specified in building codes.

## Figures and Tables

**Table 1:** Material properties used in the numerical model.

Material	Property	Proposed by
Concrete	Density	Eurocode 2 [33]
	Thermal properties—thermal conductivity and specific heat	Eurocode 2 [33]
	Mechanical properties—strength and stiffness	Stress–strain relations in Eurocode 2 [33]
	Thermal expansion	Eurocode 2 [33]
Steel rebar	Density	Eurocode 2 [33]
	Thermal properties—thermal conductivity and specific heat	Eurocode 2 [33]
	Mechanical properties—strength and stiffness	Stress–strain relations in Eurocode 2 [33]
	Thermal expansion	Eurocode 2 [33]
Fire insulation	Density	Kodur and Shakya [35]
	Thermal properties—thermal conductivity and specific heat	Kodur and Shakya [35]
FRP reinforcement	Density	Griff et al. [34]
	Thermal properties—thermal conductivity and specific heat	Griff et al. [34]
	Mechanical properties—strength and stiffness	CFRP—strength and stiffness degradation by Bisby et al. [36] Bio-based FRP—assumed strength and stiffness degradation (Figure 2)

**Table 2:** Details of RC beams and slabs with NSM FRP reinforcement.

RC Member	Steel Rebar	NSM FRP	Insulation	Load, P (kN) (Ratio *)	Fire Resistance (Min)
Beams					
B1	3 ϕ19 mm in tension 4 ϕ12 mm in compression	2 CFRP strips— 13.5 mm × 4.5 mm	None	62 (50%)	210
B2	3 ϕ19 mm in tension 4 ϕ12 mm in compression	2 CFRP strips— 13.5 mm × 4.5 mm	25 mm U-shaped	62 (50%)	No Failure
B3	3 ϕ19 mm in tension 4 ϕ12 mm in compression	5 bio-based FRP strips— 20 mm × 10 mm	None	30 (50%)	165
B4	3 ϕ19 mm in tension 4 ϕ12 mm in compression	5 bio-based FRP strips— 20 mm × 10 mm	25 mm U-shaped	30 (50%)	No Failure
Slabs					
S1	3 ϕ12 mm along the length	2 CFRP strips— 13.5 mm × 4.5 mm	None	10 (50%)	No Failure
S2	3 ϕ12 mm along the length	2 CFRP strips— 13.5 mm × 4.5 mm	25 mm on the bottom surface	10 (50%)	No Failure
S3	3 ϕ12 mm along the length	4 bio-based FRP strips— 15 mm × 5 mm	None	5 (50%)	215
S4	3 ϕ12 mm along the length	4 bio-based FRP strips— 15 mm × 5 mm	25 mm on the bottom surface	5 (50%)	No Failure

* Refers to the ratio of the applied load to the nominal capacity of the strengthened beams and slabs.

**Table 3:** Details of RC beams and slabs with EB FRP reinforcement.

RC Member	Steel Rebar	EB FRP	Insulation	Load, P (kN) (Ratio)	Fire Resistance (Min)
Beams					
B5	3 ϕ19 mm in tension 4 ϕ12 mm in compression	CFRP strip— 100 mm × 1 mm	None	64 (50%)	165
B6	3 ϕ19 mm in tension 4 ϕ12 mm in compression	CFRP strip— 100 mm × 1 mm	19 mm U-shaped	64 (50%)	No Failure
B7	3 ϕ19 mm in tension 4 ϕ12 mm in compression	Bio-based FRP strip— 150 mm × 10 mm	None	64 (50%)	140
B8	3 ϕ19 mm in tension 4 ϕ12 mm in compression	Bio-based FRP strip— 150 mm × 10 mm	19 mm U-shaped	64 (50%)	No Failure
Slabs					
S5	3 ϕ12 mm along the length	CFRP strip— 100 mm × 1 mm	None	10.5 (50%)	45
S6	3 ϕ12 mm along the length	CFRP strip— 100 mm × 1 mm	19 mm on the bottom surface	10.5 (50%)	No Failure
S7	3 ϕ12 mm along the length	Bio-based FRP strip— 150 mm × 10 mm	None	10.5 (50%)	35
S8	3 ϕ12 mm along the length	Bio-based FRP strip— 150 mm × 10 mm	19 mm on the bottom surface	10.5 (50%)	No Failure

## Data Availability

Some or all data, models, or codes that support the findings of this study are available from the corresponding author upon reasonable request.
